# Death of Dr. Zina Pitcher, of Detroit

**Published:** 1872-04

**Authors:** 


### Death of Dr. Zina Pitcher of Detroit.

It is with regret that we learn the death of this eminent member of the profession, who died at his house in Detroit on the 5th of April, at the advanced age of 75. Dr. Pitcher was a man who stood high in his profession and whose loss will be felt by his associates. The following resolutions were adopted by the profession in Detroit;

*Resolved*, That we have heard with profound grief of the death of that great and good man, our distinguished fellow-citizen and late associate, companion, friend and counselor, Dr. Zina Pitcher.

*Resolved*, That the medical profession, of which he was a member, a pillar and an ornament, have thus sustained an irreparable loss.

*Resolved*, That this city and the State of Michigan have been deprived of one of their oldest, truest and best public servants.

*Resolved*, That in token of our love for the man, our high appreciation of his personal probity and the great good to society at large which his life has fulfilled, we will attend his funeral in a body as mourners.

